# A pilot pragmatic trial of Mindfulness Based Dementia Care for care partners

**DOI:** 10.1002/alz.092704

**Published:** 2025-01-09

**Authors:** Leah R Hanson, Ella A Chrenka, Amanda A Herrmann, Meghan M JaKa, Michelle Barclay, Ymkje WJ Dioquino, Meghan O'Brien, Ann M Brombach, Laura Rice‐Oeschger

**Affiliations:** ^1^ HealthPartners Center for Memory & Aging, Saint Paul, MN USA; ^2^ HealthPartners Institute, Minneapolis, MN USA; ^3^ The Barclay Group, LLC, Minneapolis, MN USA; ^4^ Ray Dolby Brain Health Center; California Pacific Medical Center, San Francisco, CA USA; ^5^ Michigan Alzheimer’s Disease Center, University of Michigan, Ann Arbor, MI USA

## Abstract

**Background:**

Care partners (CPs) of people with dementia suffer from chronic stress impacting their mental and physical health. Mindfulness Based Stress Reduction (MBSR) has been shown to reduce depressive symptoms in CPs. Mindfulness‐Based Dementia Care (MBDC) is an adaptation of MBSR tailored to CPs. In addition to the core practices, stress related education, and attitudinal approaches to everyday stressors taught in MBSR, the mindfulness practices are linked with caregiving. In MBDC, care partnering *becomes* the practice. The primary aim of this study was to assess the feasibility, acceptability, and fidelity of implementing MBDC. The secondary aim was to determine the feasibility of obtaining pragmatic CP outcomes.

**Method:**

Care partners were referred from 3 memory care clinics plus community support groups and registered for MBDC on a centralized website. Feasibility measures included recruitment metrics, attendance, and questionnaire response rates. Care partner outcomes were included in the administrative data collection at registration and follow‐up, which included symptoms of CP depression, burden, self‐compassion, and mindful attention. Follow‐up questionnaires were emailed immediately before and after MBDC, and again after 3 months.

**Result:**

Eleven virtual MBDC courses were conducted January‐November 2023. A total of 124 CPs registered for MBDC with a mean age of 65 years (83% white, 79% female) and 62% were caring for a spouse. Eighteen CPs remained on a waiting list as no MBDC course times fit their schedule. Five CPs were found to be ineligible after registration, 8 withdrew and 9 were lost to follow‐up prior to starting MBDC. Of the 84 CPs that started MBDC, 77 completed. Attendance rates ranged from 89‐98%. Questionnaire response rates were 100% at registration, 98% pre‐MBDC and 88% post‐MBDC. Complete outcome data at all three time points was available for 53 CPs. A preliminary analysis demonstrated that over the course of MBDC, CPs had a 2.6 point larger decrease in CESD‐10 scores, measuring depressive symptoms, as compared to their own control period (p<0.01).

**Conclusion:**

This study demonstrated that MBDC was successfully implemented with fidelity including high attendance rates. However, pragmatic collection of CP outcomes through administrative data may not be adequate for an effectiveness trial.

